# Mapping of global scientific research in comorbidity and multimorbidity: A cross-sectional analysis

**DOI:** 10.1371/journal.pone.0189091

**Published:** 2018-01-03

**Authors:** Ferrán Catalá-López, Adolfo Alonso-Arroyo, Matthew J. Page, Brian Hutton, Rafael Tabarés-Seisdedos, Rafael Aleixandre-Benavent

**Affiliations:** 1 Department of Medicine, University of Valencia/INCLIVA Health Research Institute and CIBERSAM, Valencia, Spain; 2 Fundación Instituto de Investigación en Servicios de Salud, Valencia, Spain; 3 Clinical Epidemiology Program, Ottawa Hospital Research Institute, Ottawa, Ontario, Canada; 4 Department of History of Science and Documentation, University of Valencia, Valencia, Spain; 5 Unidad de Información e Investigación Social y Sanitaria-UISYS, University of Valencia and Spanish National Research Council (CSIC), Valencia, Spain; 6 School of Public Health and Preventive Medicine, Monash University, Melbourne, Australia; 7 School of Epidemiology, Public Health and Preventive Medicine, University of Ottawa, Ottawa, Ontario, Canada; 8 Ingenio-Spanish National Research Council (CSIC) and Universitat Politécnica de Valencia (UPV), Valencia, Spain; Universita degli Studi di Firenze, ITALY

## Abstract

**Background:**

The management of comorbidity and multimorbidity poses major challenges to health services around the world. Analysis of scientific research in comorbidity and multimorbidity is limited in the biomedical literature. This study aimed to map global scientific research in comorbidity and multimorbidity to understand the maturity and growth of the area during the past decades.

**Methods and findings:**

This was a cross-sectional analysis of the Web of Science. Searches were run from inception until November 8, 2016. We included research articles or reviews with no restrictions by language or publication date. Data abstraction was done by one researcher. A process of standardization was conducted by two researchers to unify different terms and grammatical variants and to remove typographical, transcription, and/or indexing errors. All potential discrepancies were resolved via discussion. Descriptive analyses were conducted (including the number of papers, citations, signatures, most prolific authors, countries, journals and keywords). Network analyses of collaborations between countries and co-words were presented. During the period 1970–2016, 85994 papers (64.0% in 2010–2016) were published in 3500 journals. There was wide diversity in the specialty of the journals, with psychiatry (16558 papers; 19.3%), surgery (9570 papers; 11.1%), clinical neurology (9275 papers; 10.8%), and general and internal medicine (7622 papers; 8.9%) the most common. PLOS One (1223 papers; 1.4%), the Journal of Affective Disorders (1154 papers; 1.3%), the Journal of Clinical Psychiatry (727 papers; 0.8%), the Journal of the American Geriatrics Society (634 papers; 0.7%) and Obesity Surgery (588 papers; 0.7%) published the largest number of papers. 168 countries were involved in the production of papers. The global productivity ranking was headed by the United States (37624 papers), followed by the United Kingdom (7355 papers), Germany (6899 papers) and Canada (5706 papers). Twenty authors who published 100 or more papers were identified; the most prolific authors were affiliated with Harvard Medical School, State University of New York Upstate Medical University, National Taiwan Normal University and China Medical University. The 50 most cited papers (“citation classics” with at least 1000 citations) were published in 20 journals, led by JAMA Psychiatry (11 papers) and JAMA (10 papers). The most cited papers provided contributions focusing on methodological aspects (e.g. Charlson Comorbidity Index, Elixhauser Comorbidity Index, APACHE prognostic system), but also important studies on chronic diseases (e.g. epidemiology of mental disorders and its correlates by the U.S. National Comorbidity Survey, Fried’s frailty phenotype or the management of obesity).

**Conclusions:**

Ours is the first analysis of global scientific research in comorbidity and multimorbidity. Scientific production in the field is increasing worldwide with research leadership of Western countries, most notably, the United States.

## Introduction

Over the last three to four decades, substantial progress has been made toward reducing mortality and extending life expectancy worldwide [1,2]. Although health seems to have improved globally, more people than ever are spending more time with functional health loss and disability [3,4]. In many countries and regions, the management of multiple chronic diseases in a given patient at the same time (the so-called, “comorbidity” or “multimorbidity”) poses major challenges to health services [5–9]. People with two or more chronic (physical or mental) diseases are more likely to have poor health outcomes, more complex clinical management and increased healthcare costs [8,9].

Analysis of scientific research in comorbidity and multimorbidity is limited in the biomedical literature [10–16]. For example, Fortin et al. [12] previously investigated the characteristics of the publications on multimorbidity (or comorbidity) and compared the number of publications on it with the number of publications on three common chronic conditions (asthma, hypertension, and diabetes). A restricted search of MEDLINE in 2002 identified 353 papers on multimorbidity and comorbidity for the period 1990–2002. The number and diversity of articles were both insufficient to provide relevant data to inform evidence-based care of people affected by multiple chronic conditions [12].

The scientific landscape has changed considerably in the subsequent years, including the launch of important initiatives for the clinical management of multiple chronic diseases [17–20], but also the proliferation of open-access journals to disseminate research findings [5,7,21–25]. Considering research is needed to increase knowledge in a changing research area, this study aimed to map global scientific research in comorbidity and multimorbidity to understand maturity and growth during the past decades.

## Methods

### Search strategy

We conducted a cross-sectional analysis of the Web of Science, Science Citation Index-Expanded (SCI-E) database, from inception to November 8^th^ 2016. The Web of Science has been considered the world’s leading taxonomic reference for citation analysis and prior to 2004, the only data source on citations available [26]. The search strategy for this study was designed by two senior health information specialists (AA-A, RA-B) and a clinical epidemiologist (FC-L), based on a previously published strategy [13]. The search strategy was constructed by using a combination of the following terms related to comorbidity and multimorbidity (see Box 1 for terminology): comorbidit* OR co-morbidit* OR multimorbidit* OR multi-morbidit* OR multidisease* OR multi-disease* OR multipatholog* OR multi-patholog* OR polimorbidit* OR poli-morbidit* OR polipatholog* OR poli-patholog* OR pluripatholog* OR pluri-patholog* (full strategy is available in S1 Table). We included two types of papers: research articles or reviews on comorbidity or multimorbidity of any type (physical or mental). Meeting abstracts, proceedings paper (journals, book-based), editorials, book chapters, corrections, retracted publications and other items (e.g. notes, news, etc…) were excluded. No restrictions in languages or publication date were applied to the database search.

**Box 1. Terminology**

The terms of “comorbidity” and “multimorbidity” are often used interchangeably. Many possible definitions and interpretations of the concepts of “comorbidity” and “multimorbidity” have been reported in the biomedical literature [9–11]. For example, Valderas and colleagues [9] reviewed the definitions of “comorbidity (and multimorbidity)” and their relationship to related constructs. A brief overview of common terms follows.

**Comorbidity**. A widely accepted definition of “comorbidity” is the occurrence or the existence of any distinct additional medical condition to an index disease [31]. In general, the role of coexisting conditions is of less importance and one does not assume an interaction between the multiple conditions. The nature of the conditions that co-occur have variously included (physical or mental) diseases, disorders, conditions, illnesses, or health problems. Comorbidity was first included as a MeSH term in 1989 [9,10].

**Multimorbidity**. Most authors define “multimorbidity” as the co-occurrence of two or more medical conditions in an individual without any reference to an index disease [6,9–11]. Therefore, in multimorbidity, no index disease is defined and all conditions (or “morbidities”) are regarded of equal importance. Multimorbidity constitutes a more generic, patient-centered concept, whereas comorbidity is an index disease-based concept [11]. At present, no MeSH term exists for multimorbidity.

Some authors have introduced other terms to describe the same or closely related concepts. Examples of alternative terms are: “multipathology”, “polymorbidity”, “polipathology”, and “pluripathology” [10,13,51].

**Case example**. Consider a 58-year-old woman with coronary artery disease, hypertension, and major depression. Her mental health professional, focusing on the major depression, would consider her coronary artery disease and hypertension as comorbidities. Her primary care physician might describe her as having multimorbidity, giving equal attention to her coronary artery disease, hypertension and major depression.

### Data extraction

For each included paper, data on the year of publication, the journal title, subject category, keywords, and the authors’ names, institutional affiliation(s), and country was downloaded online through the SCI-E from the Web of Science by one researcher (AA-A) in November 2016. A second researcher (FC-L) verified the data to minimize potential information errors. The SCI-E platform is a database that contains all the above information, including the full addresses of all authors of every paper. We also used the SCI-E to determine the extent to which each paper had been cited in the scientific peer-review literature using the “times cited” number (that is, the number of times a publication has been cited by other publications). A process of standardization was conducted by two researchers to bring together the different names of an author or country, and keywords. Specifically, one researcher (AA-A) checked the names by which an individual author appeared in two or more different forms (for example, “Ronald C. Kessler” or “Ronald Kessler” or “Ron Kessler”), using coincidence in that author’s place(s) of work as the basic criterion for normalization (for example, Harvard University, United States), and a second researcher (FC-L) verified data. We used both ‘‘author keywords” and ‘‘keyword plus,” which are automatically assigned by the Web of Science from the titles of the references of the articles because this approach has proven to be highly effective in representing the conceptual content of articles. To ensure consistency in the data, one researcher (RA-B) corrected keywords unifying grammatical variants and using only one keyword developed names of the same concept (for example, “diabetes mellitus” or “diabetes” or “adult diabetes” or “diabetes type 2” or “type 2 juvenile diabetes”). In addition, the same researcher (RA-B) removed typographical, transcription and/or indexing errors, and a second researcher (FC-L) verified data. All potential discrepancies were resolved via discussion. All these data were entered into a Microsoft Access® (Microsoft, Seattle, WA, United States) database.

### Data analysis

In this paper, we analyzed data including the number of papers, citations, signatures of authors, collaboration index (which is the mean number of author’s signatures per paper), countries, journals and keywords. Data were summarized as frequencies and percentages for categorical items. We have presented in tables the most prolific authors and countries (> 100 papers), and the most cited papers (>1000 citations). We have presented network graphs (or diagrams) to represent data visualization of the structure of the most intense scientific collaboration between countries applying a threshold of 50 papers in collaboration. In order to depict the frequency of the most frequently used keywords, a word cloud was created using Wordle (http://www.wordle.net/), which is free-software that generates “word clouds” from text that the user provides and places more emphasis on words that appear with greater frequency in the source text. We identified the most frequently used keywords per journal subject category. We also presented the “co-words network” of keywords representing the co-occurrence phenomenon of highly frequent words in the papers. The co-words network reflects the relation among multiple terms, and so is effective in mapping the associations between keywords in textual data [27]. We used Pajek [28], a software package for large network analysis that is free for non-commercial use, to construct network graphs. PRISMA checklist [29,30] (http://www.prisma-statement.org/) guided the reporting of the present analysis (and is available in S1 Checklist).

## Results

**Fig 1:**
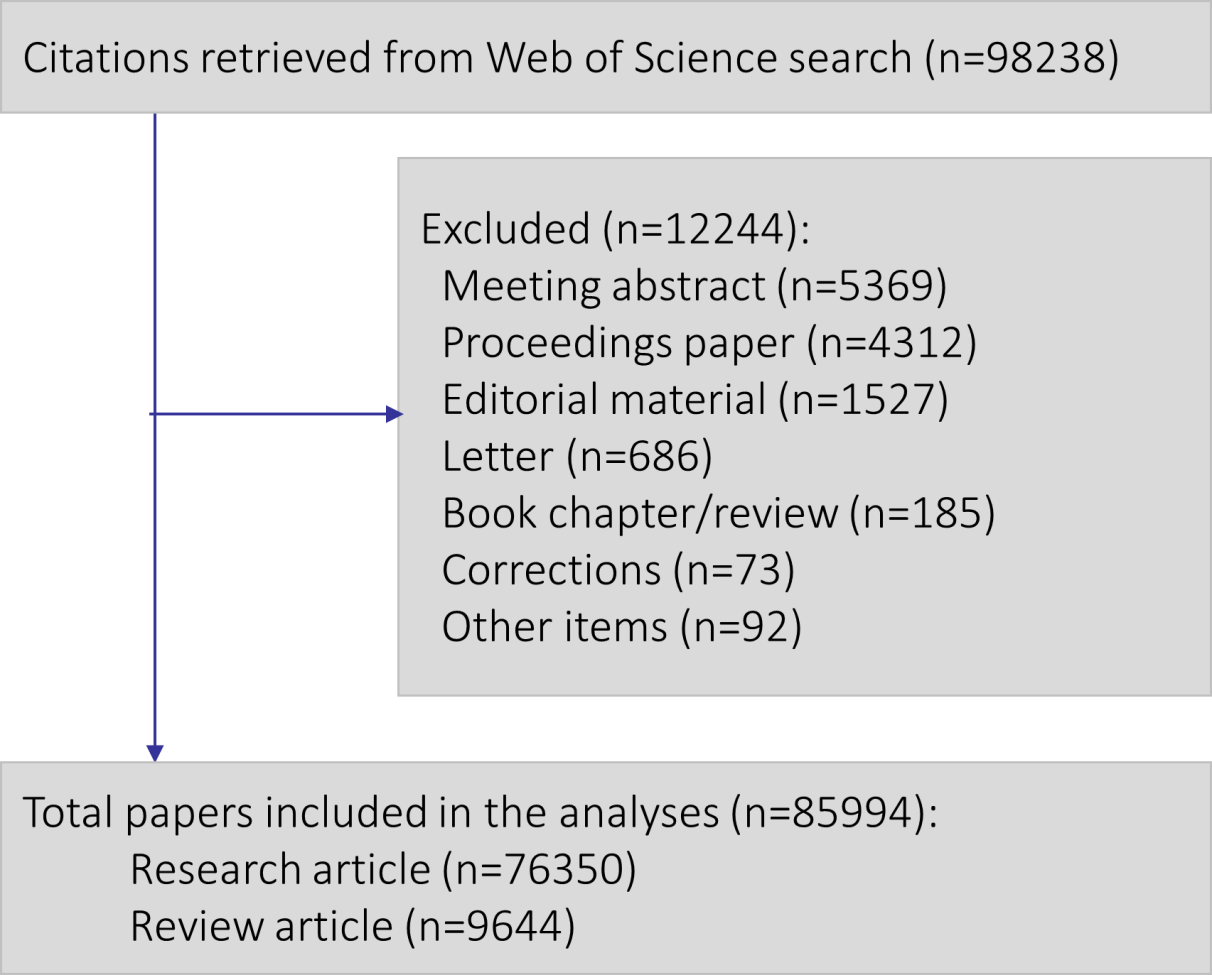
Selection of papers. Flowchart.

A total of 85994 papers (76350 articles and 9644 reviews) were identified and included in the analyses (Fig 1). Table 1 details the general characteristics of the papers.

**Table 1:** General characteristics of the sample of study.

Characteristic	Category	Number	Percent
Total number of papers		85994	100.0
Year of publication			
	1970–1979	3	0.0
	1980–1989	52	0.1
	1990–1999	4179	4.9
	2000–2009	26719	31.0
	2010–2016^a^	55041	64.0
Number of authors			
	1	4730	5.5
	2–3	18953	22.0
	4–6	35062	40.8
	7–10	20583	23.9
	>10	6666	7.8
Number of subject category^b^			
	1	50354	58.6
	2	52748	30.7
	3	7903	9.2
	4	1200	1.4
	5	163	0.2
Main subject category^b^			
	Psychiatry	16558	19.3
	Surgery	9570	11.1
	Clinical Neurology	9275	10.8
	Medicine, General & Internal	7622	8.9
	Cardiac & Cardiovascular Systems	5098	5.9
Country of first author (top-10)			
	United States	33171	38.6
	Germany	5408	6.3
	United Kingdom	4945	5.8
	Canada	4221	4.9
	Italy	4107	4.8
	Spain	3070	3.6
	Australia	2998	3.5
	The Netherlands	2766	3.2
	France	2737	3.2
	Taiwan (Republic of China)	2034	2.4

^a^November 8^th^, 2016.

^b^Subject category according to Journal Citation Report.

### Publication trend

**Fig 2:**
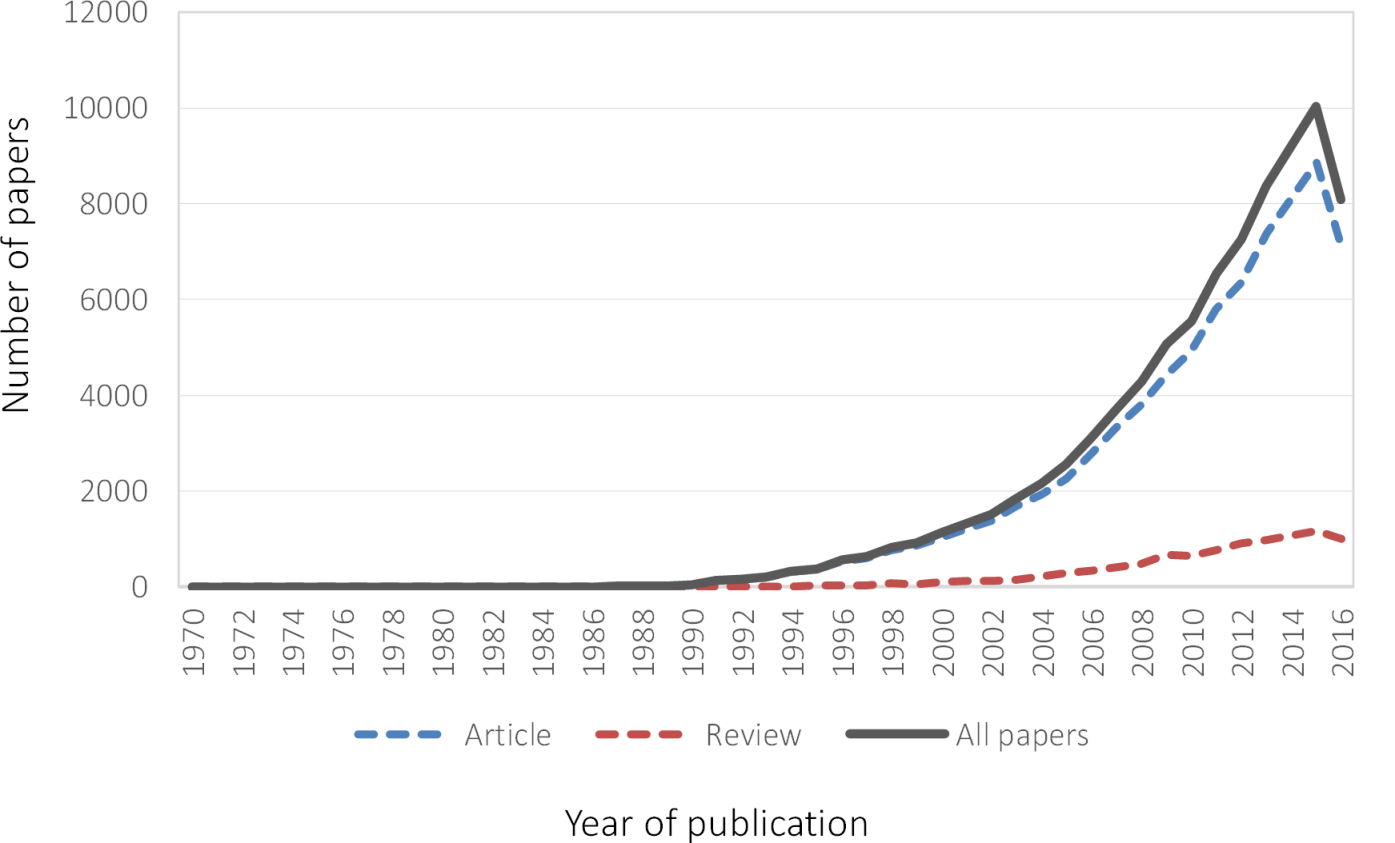
Number of papers by year of publication. Note: Data for 2016 up to November 8^th^.

The number of papers increased exponentially over the study period (Fig 2). Approximately two-thirds of the papers have been published since 2010. The first paper was published in 1970 by Prof. Alvan R. Feinstein [31] providing the seminal definition of comorbidity referring to “any distinct clinical entity that has co-existed or that may occur during the clinical course of a patient who has the index disease under study.”

### Journals and subject categories

3500 journals published 85994 papers. 596 (17.0%) journals published only one paper, 344 (9.8%) journals published two, 220 (6.3%) journals published three, and 2340 (66.8%) published four or more papers. *PLOS One* (n = 1223; 1.4%) and the *Journal of Affective Disorders* (n = 1154; 1.3%) published the largest number of papers, followed by the *Journal of Clinical Psychiatry* (n = 727; 0.8%), the *Journal of the American Geriatrics Society* (n = 634; 0.7%) and *Obesity Surgery* (n = 588; 0.7%). Most papers were classified in one (n = 50354; 58.6%) or two (n = 52748; 30.7%) journal’s subject categories. There was wide diversity in journal’s subject categories, with psychiatry, surgery, clinical neurology, and general and internal medicine the most common (Table 1).

### Authors and countries

Most papers were written by 4 or more authors (72.5%; n = 62311) and only 5.5% (n = 4730) of papers were written by one author. The first authors of the papers were based most commonly in North America and Europe; first authors from the United States were responsible for 38.6% (n = 33171) of the papers (Table 1). We identified 20 authors who published 100 or more papers (Table 2). The most prolific authors were Ronald C Kessler with 331 (from Harvard Medical School, United States), Joseph Biederman with 248 (from Harvard Medical School, United States), Stephen V Faraone with 227 (from State University of New York Upstate Medical University, United States), Chia-Hung Kao with 223 (from National Taiwan Normal University, Taiwan) and Cheng-Li Lin with 193 papers (from China Medical University, China).

**Table 2:** Most productive authors.

Author	Affiliation and country	Total papers	Total citations	Citations per paper	Papers in collaboration	Total signatures	Collaboration index (signatures per paper)
Ronald C Kessler	Harvard Medical School, United States	331	81160	245.2	324	3389	10.2
Joseph Biederman	Harvard Medical School and Massachusetts General Hospital, United States	248	19964	80.5	244	1685	6.8
Stephen V Faraone	State University of New York Upstate Medical University, United States	227	16927	74.6	226	1777	7.8
Chia-Hung Kao	National Taiwan Normal University, Taiwan (Republic of China)	223	636	2.9	223	1309	5.9
Cheng-Li Lin	China Medical University Hospital, China Medical University, China	193	550	2.8	193	1132	5.9
Murray B Stein	University of California, United States	166	10590	63.8	161	1755	10.6
Hans-Ulrich Wittchen	Dresden University of Technology, Germany	162	24910	153.8	158	1214	7.5
Henrik Toft Sørensen	Aarhus University Hospital, Denmark	162	3299	20.4	162	1022	6.3
Kenneth S Kendler	Virginia Commonwealth University, United States	160	19554	122.2	156	1748	10.9
Dan J Stein	University of Cape Town, South Africa	149	5773	38.7	147	1937	13.0
Kathleen Ries Merikangas	National Institute of Mental Health (NIMH), United States	137	15240	111.2	132	864	6.3
Fung-Chang Sung	China Medical University, China	125	977	7.8	125	888	7.1
Jitender Sareen	University of Manitoba, Canada	116	4146	35.7	115	617	5.3
Ron de Graaf	Netherlands Institute of Mental Health and Addiction (NIMHA), Netherlands	114	8051	70.6	114	1331	11.7
Hagop S Akiskal	University of California, United States	112	7029	62.8	108	748	6.7
Jordi Alonso	IMIM Hospital del Mar Medical Research Institute, Spain	111	8527	76.8	111	1717	15.5
Tzeng-Ji Chen	National Yang-Ming University, Taipei Veterans General Hospital, Taiwan (Republic of China)	109	904	8.3	109	1051	9.6
Wayne J Katon	University of Washington, United States	107	9409	87.9	103	738	6.9
Josep M Haro	Parc Sanitari Sant Joan de Déu, Spain	105	8410	80.1	105	2712	25.8
Luigi Ferrucci	National Institute on Aging (NIA), United States	104	4307	41.4	104	927	8.9

**Fig 3:**
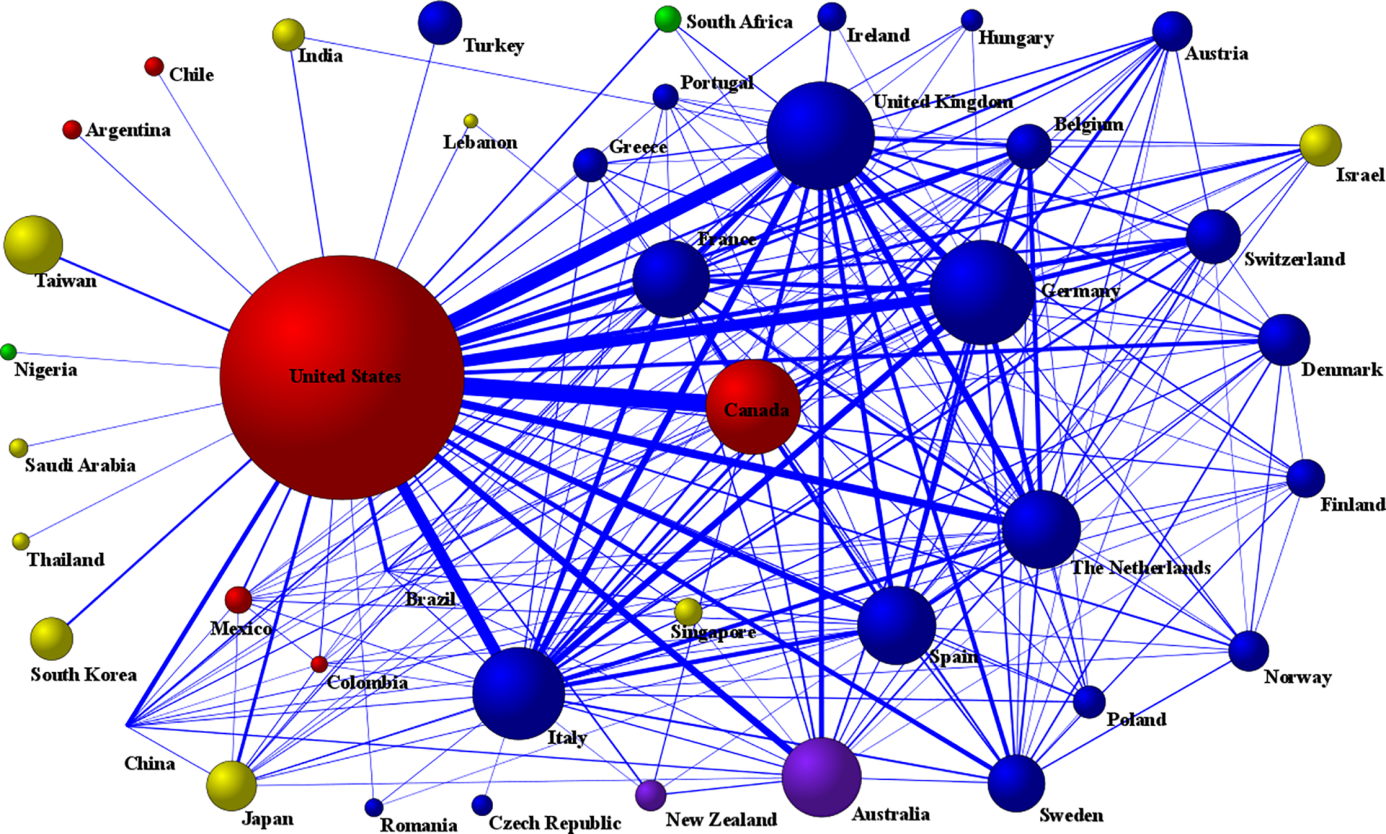
Global collaborative network between countries. Note: Most productive cluster of countries applying a threshold of 50 or more papers signed in co-authorship. Node sizes are proportional to the number of papers and line thicknesses are proportional to the number of collaborations. Node colors: America = red; Asia = yellow; Africa = green; Europe = blue; Oceania = purple.

Overall, 168 countries worldwide were involved in the sample of papers. The productivity ranking for countries with respect to the number of papers (Table 3) was headed by the United States (37624 papers), followed by the United Kingdom (7355 papers), Germany (6899 papers) and Canada (5706 papers). Fig 3 shows a visual representation of the most intense collaborative network between 42 countries (with at least 50 papers in co-authorship), in which we can see the relationships of some countries with respect to others and the position that each occupies in the network.

**Table 3:** Productivity and patterns of collaboration by 50 top countries.

Country	Total papers	Papers per million inhabitants	Total collaborations	Total citations	Citations per paper	Papers in collaboration (distinct country)	Distinct countries of collaboration	Main collaborator (and number of collaborations)
United States	37624	117.1	14296	1211072	32.2	7905	146	Canada (1574)
United Kingdom	7355	112.9	9301	186402	25.3	3669	130	United States (1336)
Germany	6899	84.7	7395	162829	23.6	2504	126	United States (999)
Canada	5706	159.2	4893	165784	29.1	2442	118	United States (1574)
Italy	5373	88.4	7094	120728	22.5	2093	122	United States (1045)
Australia	3979	167.3	3809	88132	22.2	1575	117	United States (639)
Spain	3889	83.8	5391	75487	19.4	1289	122	United States (569)
Netherlands	3885	229.4	6226	110756	28.5	1786	116	United States (728)
France	3742	56.0	5747	83846	22.4	1506	125	United States (634)
Taiwan/Republic of China	2173	92.4	756	23526	10.8	349	95	United States (218)
Sweden	2066	210.8	3486	53826	26.1	1167	114	United States (428)
Brazil	1978	9.5	2356	29773	15.1	658	119	United States (374)
Switzerland	1852	223.5	3659	56315	30.4	1190	118	Germany (454)
China	1735	1.3	2458	25828	14.9	693	111	United States (422)
Denmark	1689	297.6	2498	41416	24.5	798	110	United States (378)
Japan	1576	12.4	2156	29888	19.0	443	109	United States (313)
Belgium	1264	112.0	4036	36184	28.6	854	96	Netherlands (405)
Turkey	1203	15.3	983	10626	8.8	171	109	United States (88)
South Korea	1142	22.6	925	14698	12.9	288	103	United States (220)
Israel	1087	129.7	1952	26029	24.0	445	108	United States (310)
Norway	1024	197.1	1796	24185	23.6	520	107	United States (185)
Austria	1000	116.1	2577	23361	23.4	597	108	Germany (319)
Finland	923	168.4	1618	24740	26.8	403	110	United Kingdom (158)
Greece	738	68.2	1631	16008	21.7	375	112	United States (137)
Poland	664	17.5	1746	10806	16.3	276	83	United Kingdom (133)
India	652	0.5	954	9278	14.2	207	102	United States (112)
New Zealand	599	130.3	1362	23448	39.2	339	95	United States (168)
Ireland	513	110.5	1094	12680	24.7	276	94	United Kingdom (144)
Singapore	482	87.1	794	9344	19.4	219	108	United States (111)
Mexico	462	3.6	1836	17048	36.9	257	112	United States (207)
South Africa	437	8.0	1462	13692	31.3	316	113	United States (175)
Portugal	412	39.8	1406	8192	19.9	185	108	United Kingdom (92)
Hungary	295	30.0	996	7159	24.3	200	81	United States (93)
Czech Republic	272	25.8	838	5653	20.8	142	76	Italy (53)
Iran	272	3.4	345	3210	11.8	77	105	United States (31)
Russia	242	1.7	809	6023	24.9	102	111	United Kingdom (43)
Serbia	230	32.4	482	1904	8.3	84	106	Italy (30)
Argentina	224	5.2	834	8365	37.3	115	113	United States (78)
Chile	224	12.5	416	2120	9.5	105	84	United States (56)
Saudi Arabia	220	7.0	448	4250	19.3	145	95	United States (63)
Romania	209	10.5	1273	5228	25.0	119	107	Italy (68)
Croatia	207	49.0	349	2030	9.8	62	70	Italy (22)
Thailand	184	2.7	395	1767	9.6	119	82	United States (56)
Colombia	173	3.6	1316	7497	43.3	120	109	United States (95)
Egypt	165	1.8	391	1792	10.9	87	103	United States (27)
Nigeria	165	0.9	767	5583	33.8	70	92	United States (53)
Malaysia	146	4.8	229	1322	9.1	68	83	Australia (23)
Slovenia	138	66.9	630	2770	20.1	82	78	Germany (44)
Lebanon	121	20.7	992	6770	56.0	102	98	United States (81)
Pakistan	118	0.6	431	4854	41.1	45	108	United States (23)

top-50 countries with at least 100 papers. Country inhabitants (year 2015) obtained from the World Bank (http://data.worldbank.org/).

### Keywords

**Fig 4:**
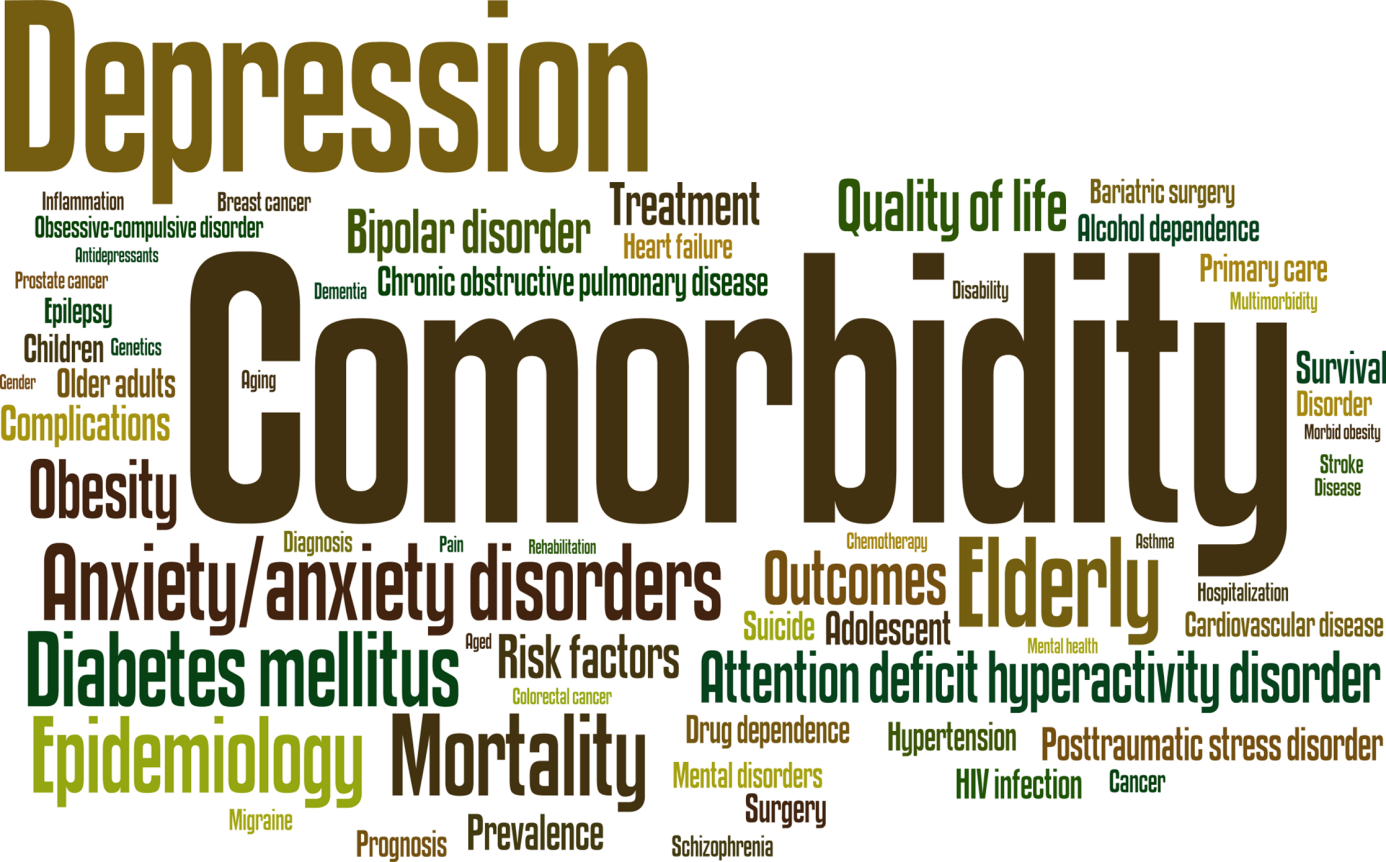
Word cloud for the frequency of terms. Note: Most frequently used keywords (at least 500 times).

**Fig 5:**
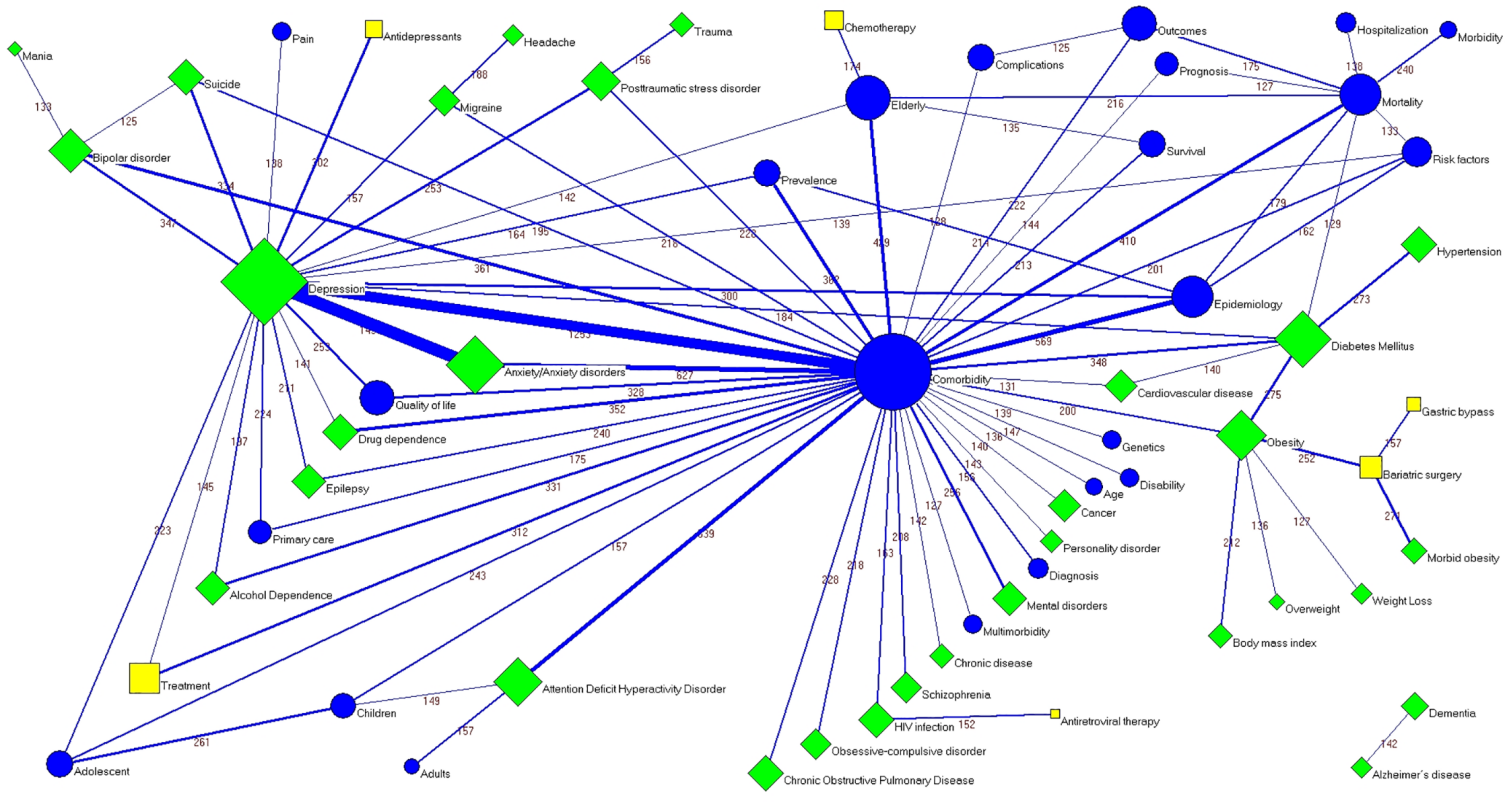
Co-words network of the author keywords. Node sizes are proportional to the number of papers and line thicknesses are proportional to the number of co-occurrences of words. Node colors: blue = words related to general terms; green = words related to diseases/disorders, signs and symptoms; yellow = interventions.

The most commonly used article/review keywords were “comorbidity” (9223 papers; 10.7%), followed by “depression” (n = 5853; 6.8%), “elderly” (n = 3077; 3.6%) and “mortality” (n = 2806; 3.3%) (Fig 4). The most frequently used keywords in the most common journal subject categories are shown in Table 4. Co-words analysis shows some associations of keywords forming triads (groupings of three terms), such as “comorbidity” and “depression” with either “anxiety/anxiety disorders”, “posttraumatic stress disorder”, “bipolar disorder”, “alcohol dependence”, “drug dependence” or “quality of life”; the associations of “diabetes mellitus” with “cardiovascular diseases”, “obesity”, or “hypertension”; and the association of “depression” with “bipolar disorder” and “suicide” (Fig 5).

**Table 4:** Most prolific journals and most commonly used keywords per journal subject category.

Journal subject category	Total papers	Journal name	Total papers		Total papers
Psychiatry	16558	Journal of Affective Disorders	1154	Depression	3290
		Journal of Clinical Psychiatry	727	Bipolar disorder	1295
		Psychiatry Research	528	Attention deficit hyperactivity disorder	1083
Surgery	9570	Obesity Surgery	588	Bariatric surgery	570
		Journal of Vascular Surgery	489	Morbid obesity	414
		Annals of Thoracic Surgery	338	Obesity	356
Clinical Neurology	9275	Journal of Affective Disorders	1154	Depression	1568
		Epilepsy & Behavior	370	Bipolar disorder	727
		Journal of Nervous and Mental Disease	365	Epilepsy	670
Medicine, General & Internal	7622	Medicine	302	Primary care	341
		Journal of General Internal Medicine	282	Depression	326
		BMJ Open	272	Diabetes mellitus	318
Cardiac & Cardiovascular Systems	5098	Annals of Thoracic Surgery	338	Heart failure	548
		American Journal of Cardiology	336	Mortality	326
		International Journal of Cardiology	269	Atrial fibrillation	226
Oncology	4790	Cancer	370	Elderly	680
		Journal of Clinical Oncology	279	Breast cancer	460
		Annals of Surgical Oncology	154	Chemotherapy	391
Neurosciences	4698	Biological Psychiatry	249	Depression	873
		Encéphale	244	Bipolar disorder	383
		Bipolar Disorders	184	Attention deficit hyperactivity disorder	311
Pharmacology & Pharmacy	4223	Drugs & Aging	236	Depression	380
		Clinical Therapeutics	134	Diabetes mellitus	173
		International Journal of Clinical Practice	125	Attention deficit hyperactivity disorder	139
Urology & Nephrology	4150	Journal of Urology	331	Mortality	327
		Nephrology Dialysis Transplantation	297	Prostate cancer	305
		Urology	247	Hemodialysis	287
Geriatrics & Gerontology	3399	Journal of the American Geriatrics Society	634	Elderly	660
		Drugs & Aging	236	Older adults	435
		Archives of Gerontology and Geriatrics	203	Depression	278
Public, Environmental & Occupational Health	3392	Medical Care	361	Depression	182
		Journal of Clinical Epidemiology	220	Diabetes mellitus	160
		BMC Public Health	220	Epidemiology	155
Respiratory System	3003	Annals of Thoracic Surgery	338	Chronic obstructive pulmonary disease	622
		Chest	282	Asthma	160
		Respiratory Medicine	185	Mortality	155
Health Care Sciences & Services	2907	Medical Care	361	Quality of life	188
		Journal of General Internal Medicine	282	Depression	154
		BMC Health Services Research	245	Diabetes mellitus	148
Psychology	2788	Psychological Medicine	517	Depression	598
		Depression and Anxiety	407	Epidemiology	166
		International Journal of Eating Disorders	249	Posttraumatic stress disorder	144
Pediatrics	2739	Journal of the American Academy of Child and Adolescent Psychiatry	376	Attention deficit hyperactivity disorder	330
		Pediatrics	167	Children	302
		Journal of Child and Adolescent Psychopharmacology	103	Adolescent	289
Endocrinology & Metabolism	2517	Diabetes Care	185	Diabetes mellitus	359
		Obesity	137	Obesity	239
		Osteoporosis International	118	Depression	122
Peripheral Vascular Disease	2446	Journal of Vascular Surgery	489	Hypertension	190
		Annals of Vascular Surgery	193	Stroke	159
		Circulation	192	Mortality	133
Psychology, Clinical	2429	Journal of Clinical Psychiatry	727	Depression	374
		Psychological Medicine	517	Anxiety disorders	130
		Depression and Anxiety	407	Eating disorders	121
Gastroenterology & Hepatology	2345	Journal of Gastrointestinal Surgery	186	Colorectal Cancer	144
		World Journal of Gastroenterology	161	Mortality	112
		Diseases of the Colon & Rectum	127	Hepatitis C	105
Orthopedics	2300	Spine	306	Mortality	123
		Clinical Orthopaedics and Related Research	195	Risk factors	96
		Journal of Bone and Joint Surgery	164	Hip fracture	95
Infectious Diseases	1792	Clinical Infectious Diseases	151	HIV infection	398
		BMC Infectious Diseases	129	Mortality	146
		Infection Control and Hospital Epidemiology	90	Bacteremia	104
Immunology	1691	Clinical Infectious Diseases	151	HIV infection	239
		Transplantation Proceedings	134	Asthma	136
		Biology of Blood and Marrow Transplantation	112	Influenza	74
Rheumatology	1610	Journal of Rheumatology	222	Rheumatoid arthritis	293
		Rheumatology	132	Osteoarthritis	91
		BMC Musculoskeletal Disorders	122	Gout	78
Gerontology	1597	Journal of the American Geriatrics Society	634	Elderly	660
		Journals of Gerontology Series A	184	Older adults	435
		International Journal of Geriatric Psychiatry	156	Depression	278
Critical Care Medicine	1589	Chest	282	Mortality	191
		Critical Care Medicine	183	Intensive care	156
		Injury	123	Chronic obstructive pulmonary disease	90

### Most cited papers

Overall, included papers received 1.9 million citations, of which 40.9% citations (n = 808817) corresponded to 3596 (4.2%) papers with at least 100 citations. The most cited papers by number of citations (“citation classics” with at least 1000 citations) are listed in Table 5. All the 50 most cited papers were published in English. These most cited articles were published in 20 journals, led by the *Archives of General Psychiatry* (now, renamed *JAMA Psychiatry*) with 11 papers and followed by the *Journal of the American Medical Association* (JAMA) with 8 papers. The list of most cited papers (Table 4) contains contributions dealing with methodological aspects, but also important epidemiological studies on chronic non-communicable diseases, comorbidity and/or multimorbidity. Some of the methodological papers present the most commonly used measures of comorbidities in health services and outcomes research: the “Charlson Comorbidity Index” [32,33] and its various adaptations (papers number-1, number-4, number-17 and number-20 in Table 5) and the “Elixhauser Comorbidity Index” [34] (paper number-10). These comorbidity index measures capture the “comorbidity burden” that exists alongside a primary diagnosis and that may influence outcomes. The list of the most cited paper also reflects major advances in the description of the epidemiology of mental disorders and its correlates by the U.S. National Comorbidity Survey [35–40] (papers number-2, number-3, number-5, number-6, number-7 and number-15, among others in Table 5); the widely-used frailty phenotype framework proposed by Fried et al. [41] (paper number-8 in Table 5)–the concepts of “frailty” and “comorbidity/multimorbidity” are commonly used interchangeably to identify vulnerable older adults [42–44], but there is a growing consensus that these might be distinct clinical entities that are causally related [42,43]; the management of predisposing factors such as obesity and overweight [45–48] (papers number-9, number-12, number-18 and number-33); the importance of hospital volume to operative mortality associated with cardiovascular and cancer procedures [49] (paper number-13 in Table 5); and the development of the widely-used “Acute Physiology and Chronic Health Evaluation (APACHE) prognostic system” [50] (paper number-14 in Table 5) to quantify the severity of illness in the intensive care units.

**Table 5:** Most cited papers.

Rank	Paper	Total citations	Citations/year
1.	Charlson ME, Pompei P, Ales KL, MacKenzie CR. A new method of classifying prognostic comorbidity in longitudinal studies: development and validation. J Chronic Dis. 1987;40:373–83.	15049	518.9
2.	Kessler RC, McGonagle KA, Zhao S, Nelson CB, Hughes M, Eshleman S, Wittchen HU, Kendler KS. Lifetime and 12-month prevalence of DSM-III-R psychiatric disorders in the United States. Results from the National Comorbidity Survey. Arch Gen Psychiatry. 1994;51:8–19.	7752	352.4
3.	Kessler RC, Berglund P, Demler O, Jin R, Merikangas KR, Walters EE. Lifetime prevalence and age-of-onset distributions of DSM-IV disorders in the National Comorbidity Survey Replication. Arch Gen Psychiatry. 2005;62:593–602.	5404	491.3
4.	Deyo RA, Cherkin DC, Ciol MA. Adapting a clinical comorbidity index for use with ICD-9-CM administrative databases. J Clin Epidemiol. 1992;45:613–9.	4463	186.0
5.	Kessler RC, Sonnega A, Bromet E, Hughes M, Nelson CB. Posttraumatic stress disorder in the National Comorbidity Survey. Arch Gen Psychiatry. 1995;52:1048–60.	4397	209.4
6.	Kessler RC, Chiu WT, Demler O, Merikangas KR, Walters EE. Prevalence, severity, and comorbidity of 12-month DSM-IV disorders in the National Comorbidity Survey Replication. Arch Gen Psychiatry. 2005;62:617–27.	4180	380.0
7.	Kessler RC, Berglund P, Demler O, Jin R, Koretz D, Merikangas KR, Rush AJ, Walters EE, Wang PS; National Comorbidity Survey Replication. The epidemiology of major depressive disorder: results from the National Comorbidity Survey Replication (NCS-R). JAMA. 2003;289:3095–105.	3476	267.4
8.	Fried LP, Tangen CM, Walston J, Newman AB, Hirsch C, Gottdiener J, Seeman T, Tracy R, Kop WJ, Burke G, McBurnie MA; Cardiovascular Health Study Collaborative Research Group. Frailty in older adults: evidence for a phenotype. J Gerontol A Biol Sci Med Sci. 2001;56:M146-56.	3052	203.5
9.	Buchwald H, Avidor Y, Braunwald E, Jensen MD, Pories W, Fahrbach K, Schoelles K. Bariatric surgery: a systematic review and meta-analysis. JAMA. 2004;292:1724–37.	2923	243.6
10.	Elixhauser A, Steiner C, Harris DR, Coffey RM. Comorbidity measures for use with administrative data. Med Care. 1998;36:8–27.	2625	145.8
11.	Regier DA, Farmer ME, Rae DS, Locke BZ, Keith SJ, Judd LL, Goodwin FK. Comorbidity of mental disorders with alcohol and other drug abuse. Results from the Epidemiologic Catchment Area (ECA) Study. JAMA. 1990;264:2511–8.	2462	94.7
12.	Must A, Spadano J, Coakley EH, Field AE, Colditz G, Dietz WH. The disease burden associated with overweight and obesity. JAMA. 1999;282:1523–9.	2409	141.7
13.	Birkmeyer JD, Siewers AE, Finlayson EV, Stukel TA, Lucas FL, Batista I, Welch HG, Wennberg DE. Hospital volume and surgical mortality in the United States. N Engl J Med. 2002;346:1128–37.	2341	167.2
14.	Knaus WA, Wagner DP, Draper EA, Zimmerman JE, Bergner M, Bastos PG, Sirio CA, Murphy DJ, Lotring T, Damiano A, et al. The APACHE III prognostic system. Risk prediction of hospital mortality for critically ill hospitalized adults. Chest. 1991;100:1619–36.	2155	86.2
15.	Kessler RC, Andrews G, Colpe LJ, Hiripi E, Mroczek DK, Normand SL, Walters EE, Zaslavsky AM. Short screening scales to monitor population prevalences and trends in non-specific psychological distress. Psychol Med. 2002;32:959–76.	2064	147.4
16.	Hoge CW, Castro CA, Messer SC, McGurk D, Cotting DI, Koffman RL. Combat duty in Iraq and Afghanistan, mental health problems, and barriers to care. N Engl J Med. 2004;351:13–22.	2013	167.8
17.	Quan H, Sundararajan V, Halfon P, Fong A, Burnand B, Luthi JC, Saunders LD, Beck CA, Feasby TE, Ghali WA. Coding algorithms for defining comorbidities in ICD-9-CM and ICD-10 administrative data. Med Care. 2005;43:1130–9.	1825	165.9
18.	Haslam DW, James WP. Obesity. Lancet. 2005 Oct 1;366(9492):1197–209.	1787	162.5
19.	DiMatteo MR, Lepper HS, Croghan TW. Depression is a risk factor for noncompliance with medical treatment: meta-analysis of the effects of anxiety and depression on patient adherence. Arch Intern Med. 2000;160:2101–7.	1525	138.6
20.	Charlson M, Szatrowski TP, Peterson J, Gold J. Validation of a combined comorbidity index. J Clin Epidemiol. 1994;47:1245–51.	1513	68.8
21.	Bousquet J, Khaltaev N, Cruz AA, Denburg J, Fokkens WJ, Togias A, Zuberbier T, Baena-Cagnani CE, Canonica GW, van Weel C, et al. Allergic Rhinitis and its Impact on Asthma (ARIA) 2008 update (in collaboration with the World Health Organization, GA(2)LEN and AllerGen). Allergy. 2008;63 Suppl 86:8–160.	1505	188.1
22.	Meyer IH. Prejudice, social stress, and mental health in lesbian, gay, and bisexual populations: conceptual issues and research evidence. Psychol Bull. 2003;129:674–97.	1501	115.5
23.	Spitzer RL, Kroenke K, Williams JB, Löwe B. A brief measure for assessing generalized anxiety disorder: the GAD-7. Arch Intern Med. 2006;166:1092–7.	1433	143.3
24.	Costello EJ, Mustillo S, Erkanli A, Keeler G, Angold A. Prevalence and development of psychiatric disorders in childhood and adolescence. Arch Gen Psychiatry. 2003;60:837–44.	1417	109.0
25.	Trivedi MH, Rush AJ, Wisniewski SR, Nierenberg AA, Warden D, Ritz L, Norquist G, Howland RH, Lebowitz B, McGrath PJ, Shores-Wilson K, et al. Evaluation of outcomes with citalopram for depression using measurement-based care in STAR*D: implications for clinical practice. Am J Psychiatry. 2006;163:28–40.	1394	139.4
26.	Vestbo J, Hurd SS, Agustí AG, Jones PW, Vogelmeier C, Anzueto A, Barnes PJ, Fabbri LM, Martinez FJ, Nishimura M, et al. Global strategy for the diagnosis, management, and prevention of chronic obstructive pulmonary disease: GOLD executive summary. Am J Respir Crit Care Med. 2013;187:347–65.	1365	455.0
27.	Hudson JI, Hiripi E, Pope HG Jr, Kessler RC. The prevalence and correlates of eating disorders in the National Comorbidity Survey Replication. Biol Psychiatry. 2007;61:348–58.	1344	149.3
28.	Kessler RC, Adler L, Barkley R, Biederman J, Conners CK, Demler O, Faraone SV, Greenhill LL, Howes MJ, Secnik K, et al. The prevalence and correlates of adult ADHD in the United States: results from the National Comorbidity Survey Replication. Am J Psychiatry. 2006;163:716–23.	1336	133.6
29.	Vos T, Flaxman AD, Naghavi M, Lozano R, Michaud C, Ezzati M, Shibuya K, Salomon JA, Abdalla S, Aboyans V, et al. Years lived with disability (YLDs) for 1160 sequelae of 289 diseases and injuries 1990–2010: a systematic analysis for the Global Burden of Disease Study 2010. Lancet. 2012;380:2163–96.	1306	326.5
30.	Ozer EJ, Best SR, Lipsey TL, Weiss DS. Predictors of posttraumatic stress disorder and symptoms in adults: a meta-analysis. Psychol Bull. 2003;129:52–73.	1282	98.6
31.	Grant BF, Stinson FS, Dawson DA, Chou SP, Dufour MC, Compton W, Pickering RP, Kaplan K. Prevalence and co-occurrence of substance use disorders and independent mood and anxiety disorders: results from the National Epidemiologic Survey on Alcohol and Related Conditions. Arch Gen Psychiatry. 2004;61:807–16.	1275	106.3
32.	Lasser K, Boyd JW, Woolhandler S, Himmelstein DU, McCormick D, Bor DH. Smoking and mental illness: A population-based prevalence study. JAMA. 2000;284:2606–10.	1275	79.7
33.	Dietz WH. Health consequences of obesity in youth: childhood predictors of adult disease. Pediatrics. 1998;101:518–25.	1271	70.6
34.	Demyttenaere K, Bruffaerts R, Posada-Villa J, Gasquet I, Kovess V, Lepine JP, Angermeyer MC, Bernert S, de Girolamo G, Morosini P, et al. Prevalence, severity, and unmet need for treatment of mental disorders in the World Health Organization World Mental Health Surveys. JAMA. 2004;291:2581–90.	1265	105.4
35.	Hagan PG, Nienaber CA, Isselbacher EM, Bruckman D, Karavite DJ, Russman PL, Evangelista A, Fattori R, Suzuki T, Oh JK, et al. The International Registry of Acute Aortic Dissection (IRAD): new insights into an old disease. JAMA. 2000;283:897–903.	1200	75.0
36.	Pories WJ, Swanson MS, MacDonald KG, Long SB, Morris PG, Brown BM, Barakat HA, deRamon RA, Israel G, Dolezal JM, et al. Who would have thought it? An operation proves to be the most effective therapy for adult-onset diabetes mellitus. Ann Surg. 1995;222:339–50.	1183	56.3
37.	Sheline YI, Wang PW, Gado MH, Csernansky JG, Vannier MW. Hippocampal atrophy in recurrent major depression. Proc Natl Acad Sci U S A. 1996;93:3908–13.	1179	59.0
38.	Kessler RC, Crum RM, Warner LA, Nelson CB, Schulenberg J, Anthony JC. Lifetime co-occurrence of DSM-III-R alcohol abuse and dependence with other psychiatric disorders in the National Comorbidity Survey. Arch Gen Psychiatry. 1997;54:313–21.	1142	60.1
39.	Weissman MM, Bland RC, Canino GJ, Faravelli C, Greenwald S, Hwu HG, Joyce PR, Karam EG, Lee CK, Lellouch J, et al. Cross-national epidemiology of major depression and bipolar disorder. JAMA. 1996;276:293–9.	1133	56.7
40.	Kessler RC, Borges G, Walters EE. Prevalence of and risk factors for lifetime suicide attempts in the National Comorbidity Survey. Arch Gen Psychiatry. 1999;56:617–26.	1112	65.4
41.	Kessler RC, Barker PR, Colpe LJ, Epstein JF, Gfroerer JC, Hiripi E, Howes MJ, Normand SL, Manderscheid RW, Walters EE, et al. Screening for serious mental illness in the general population. Arch Gen Psychiatry. 2003;60:184–9.	1108	85.2
42.	Blazer DG, Kessler RC, McGonagle KA, Swartz MS. The prevalence and distribution of major depression in a national community sample: the National Comorbidity Survey. Am J Psychiatry. 1994;151:979–86.	1096	49.8
43.	Wang PS, Lane M, Olfson M, Pincus HA, Wells KB, Kessler RC. Twelve-month use of mental health services in the United States: results from the National Comorbidity Survey Replication. Arch Gen Psychiatry. 2005;62:629–40.	1093	99.4
44.	Sullivan PF, Neale MC, Kendler KS. Genetic epidemiology of major depression: review and meta-analysis. Am J Psychiatry. 2000;157:1552–62.	1089	68.1
45.	Kessler RC, McGonagle KA, Swartz M, Blazer DG, Nelson CB. Sex and depression in the National Comorbidity Survey. I: Lifetime prevalence, chronicity and recurrence. J Affect Disord. 1993;29:85–96.	1076	46.8
46.	Moussavi S, Chatterji S, Verdes E, Tandon A, Patel V, Ustun B. Depression, chronic diseases, and decrements in health: results from the World Health Surveys. Lancet. 2007;370:851–8.	1065	118.3
47.	Romano PS, Roos LL, Jollis JG. Adapting a clinical comorbidity index for use with ICD-9-CM administrative data: differing perspectives. J Clin Epidemiol. 1993;46:1075–9.	1061	46.1
48.	Browning JD, Horton JD. Molecular mediators of hepatic steatosis and liver injury. J Clin Invest. 2004;114:147–52.	1032	86.0
49	Bair MJ, Robinson RL, Katon W, Kroenke K. Depression and pain comorbidity: a literature review. Arch Intern Med. 2003;163:2433–45.	1022	78.6
50.	Hasin DS, Stinson FS, Ogburn E, Grant BF. Prevalence, correlates, disability, and comorbidity of DSM-IV alcohol abuse and dependence in the United States: results from the National Epidemiologic Survey on Alcohol and Related Conditions. Arch Gen Psychiatry. 2007;64:830–42.	1004	111.6

Most cited (top-50) papers with at least 1000 citations.

## Discussion

In this cross-sectional analysis, we analyzed the global scientific research in comorbidity and multimorbidity for the period 1970–2016. We have identified the most productive investigators and countries, most common subjects and keywords, most prolific journals and “citation classics” in comorbidity and multimorbidity based on publications in multiple specialties and disciplines. The most striking results are the increasing number of published articles in recent years, with approximately two-thirds of the papers published since 2010. To the best of our knowledge, this is the first comprehensive global mapping analysis of scientific publications in comorbidity and multimorbidity. This analysis complements and expands the perspective of previous studies that analyzed some characteristics of articles in comorbidity [10,12], the diversity of terms used in the literature referring to the presence of multiple concurrent diseases [10,12,51], or reviews on the implications and the understanding of research needs and treatment impact [52,53].

In line with previous research in other areas [54–57], the global productivity of scientific papers is dominated by the United States (as a central hub of knowledge), followed by other nodes in Western Europe (such as the United Kingdom, Germany and Italy) and Canada. The large number of publications on comorbidity and multimorbidity from these countries reflects the importance that Western societies devote to research as the basis for socio-economic and technological development, but also reflects the interest in understanding and addressing important challenges of population aging and increased complexity of chronicity. Ageing of the world's population is increasing the number of people living with sequelae of multiple diseases, with an increasing trend in low-income countries [1–4]. As might be expected, the scientific community captured is centered on a nucleus of scientists and researchers from academia, medical and health research centers from North America and Western Europe, but also from Australia and Taiwan (Republic of China). Specifically, the most intense global collaborations took place between authors and institutions from the United States, the United Kingdom and Canada. Perhaps, the very limited participation of low and middle income-based researchers and institutions in research on comorbidity and multimorbidity could warrant further pragmatic action given that the epidemiological transition (e.g. replacement of infectious diseases by chronic diseases) imposes more constraints to deal with the burden of multiple chronic diseases in a poor environment characterized by ill-health systems [3,4,58–60].

Papers on comorbidity and multimorbidity were published most often in journals devoted to neuropsychiatry and neurosciences. Psychiatry has become one of the fastest growing medical disciplines [61]. In fact, the publication activity and interest of comorbidity and multimorbidity in people with mental disorders seems to be increasing [62,63]. Our analysis revealed that nearly 20% of all scientific production was published in journals belonging to psychiatry and mental health. This large relative productivity in psychiatry may be explained by the important role of comorbidity and its implications for theories of etiology, prevention and treatment of mental disorders [63]. Within psychiatry, comorbidity has been traditionally used to refer to the overlap of two or more psychiatric disorders [64]. Similarly, comorbidity (and multimorbidity) between mental disorders and substance use disorders [65–67], cardiovascular diseases [68–70], cancer [71,72] or other chronic disorders [6,73] has gained prominence within the past few decades.

Our analyses suggest that other medical disciplines with a large number of papers on comorbidity and multimorbidity, including surgery [74,75], clinical neurology [76–78], general and internal medicine [3,4,79,80], cardiology [81] and oncology [82], focus on those conditions with a high global burden of disease. The subject analysis has revealed that the keywords’ prioritization in comorbidity and multimorbidity depends on the addressed subject area. For example, “Depression” is the most commonly used keyword in the subject categories of Psychiatry, Clinical Neurology, Neurosciences, Psychology; but also in General and Internal Medicine, Geriatrics and Gerontology, Pharmacology, Endocrinology, Public, Environmental and Occupational Health, and Health Care Sciences and Services. Depression is a common mental disorder that occurs in people of all ages across all world regions and represents a leading cause of disease burden [3,4,78]. Despite existing evidence of the effectiveness of multiple interventions, traditional approaches to the management of the depressive disorders (such as medication alone and brief psychotherapy) have contributed to large treatment gaps [83–86]. In this respect, the complex pathogenesis implicates factors of diverse nature that should be considered in research. For example, integrating the management of depressive disorders with other common mental disorders (e.g. anxiety disorders and bipolar disorder) or other chronic conditions (e.g. cancer, diabetes and diseases of the cardiorespiratory system) through transdiagnostic interventions [83].

The topic analysis of the most cited papers (“citation classics”) allowed us to determine which topics have attracted the most interest in the research on comorbidity and multimorbidity. These include landmark methodological developments in measuring comorbidity (such as Charlson’s index, Elixhauser’s index and their modifications) [32–34] and descriptive epidemiological studies measuring the burden of comorbidity [35–40], among others. However, important knowledge gaps in comorbidity and multimorbidity remain. The limitations of clinical practice guidelines and treatment for single diseases are well recognized in the biomedical literature, along with the call to make better use of the best evidence base [87,88]. Clinical trials are usually conducted in homogeneous populations, which prevents us from knowing whether treatment effects in people with multiple chronic diseases are equivalent to those in patients with single diseases [89,90]. The evidence base for interventions to improve outcomes for people with multimorbidity therefore remains limited; however, emerging evidence is being generated to support disease management policies in primary care and community settings [53]. The consideration of people with multimorbidity is essential in future study design and evaluations of health services and technologies. To be of value, it is important that research includes the evaluation of the benefits of multiple approaches for multiple coexisting diseases (e.g. patient-, family- and population-centered), and that generalizability and applicability problems be explicitly addressed [87–91].

There are several limitations to our study. We characterized knowledge structures generated by papers included in the Web of Science database, integrating subject categories of journals, keywords of papers and network analyses. However, these methods represent a scoping approach which could be complemented further by more detailed analyses, for example analyzing the content and reporting quality of papers in evidence syntheses (including systematic reviews of the literature [92]). We only analyzed research articles and review articles. Undoubtedly, there are other important reports (e.g. health policy reports, meeting abstracts and letters/correspondence [93]) that also merit consideration in global debates and discussions in comorbidity and multimorbidity. The validity of keywords mapping and the results of the co-word analyses depend on the definitions of words chosen to conceptualize the papers by the authors or database indexers to categorize papers. The growing interest in comorbidity and multimorbidity by health care providers has resulted in more research on these issues, which may have led to a proliferation of different terms for the same concepts. For example, the traditional (and widely accepted) term “comorbidity” is associated with high volume of papers but may lack specificity, whereas the more recently introduced term of “multimorbidity” is associated with low number of papers (see S1 Table). As Almirall and Fortin stated “[t]he use of clearly defined terms in the literature is recommended until a general consensus on the terminology of multiple coexistent diseases is reached” across multiple disciplines [51]. Given the dynamic nature of the field, it will be interesting to see whether the growth trend remains in the coming years, and how the characteristics of the field changes of time (e.g. by means of longitudinal network analyses).

## Conclusion

The global analysis presented in this study provides compelling evidence of the scientific growth of research on comorbidity and multimorbidity. Scientific research in this field is increasingly published in biomedical journals, with research leadership of Western countries, most notably, the United States. This study contributes to a better understanding in this challenging field and identifies the main areas of research, the publication sources chosen for their scientific dissemination and the major scientific leaders. Advances in several subjects and research areas will allow for use of new theories and models to fundamentally changes in the management of people with multiple chronic diseases.

## Supporting information

S1 Checklist Reporting checklist. (DOCX)

S1 Table Search strategy and results. (DOCX)

## References

[1] GBD 2016 Causes of Death Collaborators. Global, regional, and national age-sex specific mortality for 264 causes of death, 1980–2016: a systematic analysis for the Global Burden of Disease Study 2016. Lancet. 2017;390(10100):1151–1210. doi: 10.1016/S0140-6736(17)32152-9 28919116 10.1016/S0140-6736(17)32152-9 PMC5605883

[2] GBD 2016 Mortality Collaborators. Global, regional, and national under-5 mortality, adult mortality, age-specific mortality, and life expectancy, 1970–2016: a systematic analysis for the Global Burden of Disease Study 2016. Lancet. 2017;390(10100):1084–1150. doi: 10.1016/S0140-6736(17)31833-0 28919115 10.1016/S0140-6736(17)31833-0 PMC5605514

[3] GBD 2016 Disease and Injury Incidence and Prevalence Collaborators. Global, regional, and national incidence, prevalence, and years lived with disability for 328 diseases and injuries for 195 countries, 1990–2016: a systematic analysis for the Global Burden of Disease Study 2016. Lancet. 2017;390(10100):1211–1259. doi: 10.1016/S0140-6736(17)32154-2 28919117 10.1016/S0140-6736(17)32154-2 PMC5605509

[4] GBD 2016 DALYs and HALE Collaborators. Global, regional, and national disability-adjusted life-years (DALYs) for 333 diseases and injuries and healthy life expectancy (HALE) for 195 countries and territories, 1990–2016: a systematic analysis for the Global Burden of Disease Study 2016. Lancet. 2017;390(10100):1260–1344. doi: 10.1016/S0140-6736(17)32130-X 28919118 10.1016/S0140-6736(17)32130-X PMC5605707

[5] Holden L, Scuffham PA, Hilton MF, Muspratt A, Ng SK, Whiteford HA. Patterns of multimorbidity in working Australians. Popul Health Metr. 2011;9(1):15 doi: 10.1186/1478-7954-9-15 21635787 10.1186/1478-7954-9-15 PMC3123553

[6] Barnett K, Mercer SW, Norbury M, Watt G, Wyke S, Guthrie B. Epidemiology of multimorbidity and implications for health care, research, and medical education: a cross-sectional study. Lancet. 2012;380(9836):37–43. doi: 10.1016/S0140-6736(12)60240-2 22579043 10.1016/S0140-6736(12)60240-2

[7] Wang HH, Wang JJ, Wong SY, Wong MC, Li FJ, Wang PX, et al Epidemiology of multimorbidity in China and implications for the healthcare system: cross-sectional survey among 162,464 community household residents in southern China. BMC Med. 2014;12:188 doi: 10.1186/s12916-014-0188-0 25338506 10.1186/s12916-014-0188-0 PMC4212117

[8] Wallace E, Salisbury C, Guthrie B, Lewis C, Fahey T, Smith SM. Managing patients with multimorbidity in primary care. BMJ. 2015;350:h176 doi: 10.1136/bmj.h176 25646760 10.1136/bmj.h176

[9] Valderas JM, Starfield B, Sibbald B, Salisbury C, Roland M. Defining comorbidity: implications for understanding health and health services. Ann Fam Med. 2009;7(4):357–63. doi: 10.1370/afm.983 19597174 10.1370/afm.983 PMC2713155

[10] van den Akker M, Buntinx F, Knottnerus J. Comorbidity or multimorbidity: what's in a name. A review of literature. Eur J Gen Pract. 1996;2:65–70. doi: 10.1016/S0895-4356(97)00306-5 PMID: 9619963

[11] Radner H, Yoshida K, Smolen JS, Solomon DH. Multimorbidity and rheumatic conditions-enhancing the concept of comorbidity. Nat Rev Rheumatol. 2014;10(4):252–6. doi: 10.1038/nrrheum.2013.212 24418765 10.1038/nrrheum.2013.212

[12] Fortin M, Lapointe L, Hudon C, Vanasse A. Multimorbidity is common to family practice: is it commonly researched? Can Fam Physician. 2005;51:244–5. 16926936 PMC1472978

[13] Smith SM, Soubhi H, Fortin M, Hudon C, O'Dowd T. Managing patients with multimorbidity: systematic review of interventions in primary care and community settings. BMJ. 2012;345:e5205 doi: 10.1136/bmj.e5205 22945950 10.1136/bmj.e5205 PMC3432635

[14] Tran VT, Montori VM, Eton DT, Baruch D, Falissard B, Ravaud P. Development and description of measurement properties of an instrument to assess treatment burden among patients with multiple chronic conditions. BMC Med. 2012;10:68 doi: 10.1186/1741-7015-10-68 22762722 10.1186/1741-7015-10-68 PMC3402984

[15] Prados-Torres A, Calderón-Larrañaga A, Hancco-Saavedra J, Poblador-Plou B, van den Akker M. Multimorbidity patterns: a systematic review. J Clin Epidemiol. 2014;67(3):254–66. doi: 10.1016/j.jclinepi.2013.09.021 24472295 10.1016/j.jclinepi.2013.09.021

[16] Muth C, Kirchner H, van den Akker M, Scherer M, Glasziou PP. Current guidelines poorly address multimorbidity: pilot of the interaction matrix method. J Clin Epidemiol. 2014;67:1242–1250. doi: 10.1016/j.jclinepi.2014.07.004 25216898 10.1016/j.jclinepi.2014.07.004

[17] U.S. Department of Health & Human Services. Multiple chronic conditions: a strategic framework. Optimum health and quality of life for individuals with multiple chronic conditions Washington, DC: US Department of Health & Human Services; 2010.

[18] Muth C, van den Akker M, Blom JW, Mallen CD, Rochon J, Schellevis FG, et al The Ariadne principles: how to handle multimorbidity in primary care consultations. BMC Med. 2014;12:223 doi: 10.1186/s12916-014-0223-1 25484244 10.1186/s12916-014-0223-1 PMC4259090

[19] Onder G, Palmer K, Navickas R, Jurevičienė E, Mammarella F, Strandzheva M, et al; Joint Action on Chronic Diseases and Promoting Healthy Ageing across the Life Cycle (JA-CHRODIS). Time to face the challenge of multimorbidity. A European perspective from the joint action on chronic diseases and promoting healthy ageing across the life cycle (JA-CHRODIS). Eur J Intern Med. 2015;26(3):157–9. doi: 10.1016/j.ejim.2015.02.020 25797840 10.1016/j.ejim.2015.02.020

[20] Palmer K, Marengoni A, Forjaz MJ, Jureviciene E, Laatikainen T, Mammarella F, et al; Joint Action on Chronic Diseases and Promoting Healthy Ageing Across the Life Cycle (JA-CHRODIS). Multimorbidity care model: Recommendations from the consensus meeting of the Joint Action on Chronic Diseases and Promoting Healthy Ageing across the Life Cycle (JA-CHRODIS). Health Policy. 2017 9 14 pii: S0168-8510(17)30234-8. doi: 10.1016/j.healthpol.2017.09.006 [Epub ahead of print] 28967492 10.1016/j.healthpol.2017.09.006

[21] Holzer BM, Siebenhuener K, Bopp M, Minder CE. Evidence-based design recommendations for prevalence studies on multimorbidity: improving comparability of estimates. Popul Health Metr. 2017;15(1):9 doi: 10.1186/s12963-017-0126-4 28270157 10.1186/s12963-017-0126-4 PMC5341353

[22] Fortin Y, Crispo JA, Cohen D, McNair DS, Mattison DR, Krewski D. External validation and comparison of two variants of the Elixhauser comorbidity measures for all-cause mortality. PLoS One. 2017;12(3):e0174379 doi: 10.1371/journal.pone.0174379 28350807 10.1371/journal.pone.0174379 PMC5369776

[23] Mondor L, Maxwell CJ, Hogan DB, Bronskill SE, Gruneir A, Lane NE, et al Multimorbidity and healthcare utilization among home care clients with dementia in Ontario, Canada: A retrospective analysis of a population-based cohort. PLoS Med. 2017;14(3):e1002249 doi: 10.1371/journal.pmed.1002249 28267802 10.1371/journal.pmed.1002249 PMC5340355

[24] Stubbs B, Koyanagi A, Veronese N, Vancampfort D, Solmi M, Gaughran F, et al Physical multimorbidity and psychosis: comprehensive cross sectional analysis including 242,952 people across 48 low- and middle-income countries. BMC Med. 2016;14(1):189 doi: 10.1186/s12916-016-0734-z 27871281 10.1186/s12916-016-0734-z PMC5118890

[25] Valderas JM, Mercer SW, Fortin M. Research on patients with multiple health conditions: different constructs, different views, one voice. J Comorb. 2011;1:1–3. doi: 10.15256/joc.2011.1.11 29090129 10.15256/joc.2011.1.11 PMC5556414

[26] Harzing AW, Alakangas S. Google Scholar, Scopus and the Web of Science: a longitudinal and cross-disciplinary comparison. Scientometrics. 2016;106:787–804. doi: 10.1007/s11192-015-1798-9

[27] Aleixandre JL, Aleixandre-Tudó JL, Bolaños-Pizzaro M, Aleixandre-Benavent R. Mapping the scientific research on wine and health (2001–2011). J Agric Food Chem. 2013;61:11871–80. doi: 10.1021/jf404394e 24274031 10.1021/jf404394e

[28] Batagelj V, Mrvar A: Pajek 1.28. Program for large network analysis. 2010, Ljubljana: University of Ljubljana.

[29] Moher D, Liberati A, Tetzlaff J, Altman DG; PRISMA Group. Preferred reporting items for systematic reviews and meta-analyses: the PRISMA statement. PLoS Med. 2009;6(7):e1000097 doi: 10.1371/journal.pmed.1000097 19621072 10.1371/journal.pmed.1000097 PMC2707599

[30] Liberati A, Altman DG, Tetzlaff J, Mulrow C, Gøtzsche PC, Ioannidis JP, et al The PRISMA statement for reporting systematic reviews and meta-analyses of studies that evaluate health care interventions: explanation and elaboration. PLoS Med. 2009;6(7):e1000100 doi: 10.1371/journal.pmed.1000100 19621070 10.1371/journal.pmed.1000100 PMC2707010

[31] Feinstein AR. Pre-therapeutic classification of co-morbidity in chronic disease. J Chronic Dis. 1970;23:455–468. doi: 10.1016/0021-9681(70)90054-8 26309916 10.1016/0021-9681(70)90054-8

[32] Charlson ME, Pompei P, Ales KL, MacKenzie CR. A new method of classifying prognostic comorbidity in longitudinal studies: development and validation. J Chronic Dis. 1987;40:373–83. doi: 10.1016/0021-9681(87)90171-8 3558716 10.1016/0021-9681(87)90171-8

[33] Charlson M, Szatrowski TP, Peterson J, Gold J. Validation of a combined comorbidity index. J Clin Epidemiol. 1994;47:1245–51. doi: 10.1016/0895-4356(94)90129-5 7722560 10.1016/0895-4356(94)90129-5

[34] Elixhauser A, Steiner C, Harris DR, Coffey RM. Comorbidity measures for use with administrative data. Med Care. 1998;36:8–27. 9431328 10.1097/00005650-199801000-00004

[35] Kessler RC, McGonagle KA, Zhao S, Nelson CB, Hughes M, Eshleman S, et al Lifetime and 12-month prevalence of DSM-III-R psychiatric disorders in the United States. Results from the National Comorbidity Survey. Arch Gen Psychiatry. 1994;51:8–19. doi: 10.1001/archpsyc.1994.03950010008002 8279933 10.1001/archpsyc.1994.03950010008002

[36] Kessler RC, Berglund P, Demler O, Jin R, Merikangas KR, Walters EE. Lifetime prevalence and age-of-onset distributions of DSM-IV disorders in the National Comorbidity Survey Replication. Arch Gen Psychiatry. 2005;62:593–602. doi: 10.1001/archpsyc.62.6.593 15939837 10.1001/archpsyc.62.6.593

[37] Kessler RC, Sonnega A, Bromet E, Hughes M, Nelson CB. Posttraumatic stress disorder in the National Comorbidity Survey. Arch Gen Psychiatry. 1995;52:1048–60. doi: 10.1001/archpsyc.1995.03950240066012 7492257 10.1001/archpsyc.1995.03950240066012

[38] Kessler RC, Chiu WT, Demler O, Merikangas KR, Walters EE. Prevalence, severity, and comorbidity of 12-month DSM-IV disorders in the National Comorbidity Survey Replication. Arch Gen Psychiatry. 2005;62:617–27. doi: 10.1001/archpsyc.62.6.617 15939839 10.1001/archpsyc.62.6.617 PMC2847357

[39] Kessler RC, Berglund P, Demler O, Jin R, Koretz D, Merikangas KR, et al; National Comorbidity Survey Replication. The epidemiology of major depressive disorder: results from the National Comorbidity Survey Replication (NCS-R). JAMA. 2003;289:3095–105. doi: 10.1001/jama.289.23.3095 12813115 10.1001/jama.289.23.3095

[40] Kessler RC, Andrews G, Colpe LJ, Hiripi E, Mroczek DK, Normand SL, et al Short screening scales to monitor population prevalences and trends in non-specific psychological distress. Psychol Med. 2002;32:959–76. doi: 10.1017/S0033291702006074 12214795 10.1017/s0033291702006074

[41] Fried LP, Tangen CM, Walston J, Newman AB, Hirsch C, Gottdiener J, et al; Cardiovascular Health Study Collaborative Research Group. Frailty in older adults: evidence for a phenotype. J Gerontol A Biol Sci Med Sci. 2001;56:M146–56. doi: 10.1093/gerona/56.3.M146 11253156 10.1093/gerona/56.3.m146

[42] Fried LP, Ferrucci L, Darer J, Williamson JD, Anderson G. Untangling the concepts of disability, frailty, and comorbidity: implications for improved targeting and care. J Gerontol A Biol Sci Med Sci. 2004;59:255–63. doi: 10.1093/gerona/59.3.M255 15031310 10.1093/gerona/59.3.m255

[43] Rodríguez-Mañas L, Féart C, Mann G, Viña J, Chatterji S, Chodzko-Zajko W, et al Searching for an operational definition of frailty: a Delphi method based consensus statement: the frailty operative definition-consensus conference project. J Gerontol A Biol Sci Med Sci. 2013;68(1):62–7. doi: 10.1093/gerona/gls119 22511289 10.1093/gerona/gls119 PMC3598366

[44] Hopman P, de Bruin SR, Forjaz MJ, Rodriguez-Blazquez C, Tonnara G, Lemmens LC, et al Effectiveness of comprehensive care programs for patients with multiple chronic conditions or frailty: A systematic literature review. Health Policy. 2016;120:818–32. doi: 10.1016/j.healthpol.2016.04.002 27114104 10.1016/j.healthpol.2016.04.002

[45] Buchwald H, Avidor Y, Braunwald E, Jensen MD, Pories W, Fahrbach K, Schoelles K. Bariatric surgery: a systematic review and meta-analysis. JAMA. 2004;292:1724–37. doi: 10.1001/jama.292.14.1724 15479938 10.1001/jama.292.14.1724

[46] Must A, Spadano J, Coakley EH, Field AE, Colditz G, Dietz WH. The disease burden associated with overweight and obesity. JAMA. 1999;282:1523–9. doi: 10.1001/jama.282.16.1523 10546691 10.1001/jama.282.16.1523

[47] Haslam DW, James WP. Obesity. Lancet. 2005;366(9492):1197–209. doi: 10.1016/S0140-6736(05)67483-1 16198769 10.1016/S0140-6736(05)67483-1

[48] Dietz WH. Health consequences of obesity in youth: childhood predictors of adult disease. Pediatrics. 1998;101:518–25. 12224658

[49] Birkmeyer JD, Siewers AE, Finlayson EV, Stukel TA, Lucas FL, Batista I, et al Hospital volume and surgical mortality in the United States. N Engl J Med. 2002;346:1128–37. doi: 10.1056/NEJMsa012337 11948273 10.1056/NEJMsa012337

[50] Knaus WA, Wagner DP, Draper EA, Zimmerman JE, Bergner M, Bastos PG, et al The APACHE III prognostic system. Risk prediction of hospital mortality for critically ill hospitalized adults. Chest. 1991;100:1619–36. doi: 10.1378/chest.100.6.1619 1959406 10.1378/chest.100.6.1619

[51] Almirall J, Fortin M. The coexistence of terms to describe the presence of multiple concurrent diseases. J Comorb. 2013;3:4–9. doi: 10.15256/joc.2013.3.22 29090140 10.15256/joc.2013.3.22 PMC5636023

[52] Boyd CM, Fortin M. Future of multimorbidity research: How should understanding of multimorbidity inform health system design? Public Health Reviews. 2010;32(2):451–74. doi: 10.1007/BF03391611

[53] Smith SM, Wallace E, O'Dowd T, Fortin M. Interventions for improving outcomes in patients with multimorbidity in primary care and community settings. Cochrane Database Syst Rev. 2016;3:CD006560 doi: 10.1002/14651858.CD006560.pub3 26976529 10.1002/14651858.CD006560.pub3 PMC6703144

[54] Wagstaff A, Culyer AJ. Four decades of health economics through a bibliometric lens. J Health Econ. 2012;31(2):406–39. doi: 10.1016/j.jhealeco.2012.03.002 22481096 10.1016/j.jhealeco.2012.03.002

[55] Boyack KW, Klavans R, Sorensen AA, Ioannidis JP. A list of highly influential biomedical researchers, 1996–2011. Eur J Clin Invest. 2013;43(12):1339–65. doi: 10.1111/eci.12171 24134636 10.1111/eci.12171

[56] Catalá-López F, Alonso-Arroyo A, Hutton B, Aleixandre-Benavent R, Moher D. Global collaborative networks on meta-analyses of randomized trials published in high impact factor medical journals: a social network analysis. BMC Med. 2014;12:15 doi: 10.1186/1741-7015-12-15 24476131 10.1186/1741-7015-12-15 PMC3913337

[57] Haunschild R, Bornmann L, Marx W. Climate Change Research in View of Bibliometrics. PLoS One. 2016;11(7):e0160393 doi: 10.1371/journal.pone.0160393 27472663 10.1371/journal.pone.0160393 PMC4966958

[58] Bollyky TJ, Templin T, Cohen M, Dieleman JL. Lower-income countries that face the most rapid shift in noncommunicable disease burden are also the least prepared. Health Aff (Millwood). 2017;36(11):1866–1875. doi: 10.1377/hlthaff.2017.0708 29137514 10.1377/hlthaff.2017.0708 PMC7705176

[59] Global Burden of Disease Health Financing Collaborator Network. Future and potential spending on health 2015–40: development assistance for health, and government, prepaid private, and out-of-pocket health spending in 184 countries. Lancet. 2017;389(10083):2005–2030. doi: 10.1016/S0140-6736(17)30873-5 28433260 10.1016/S0140-6736(17)30873-5 PMC5440765

[60] Global Burden of Disease Health Financing Collaborator Network. Evolution and patterns of global health financing 1995–2014: development assistance for health, and government, prepaid private, and out-of-pocket health spending in 184 countries. Lancet. 2017;389(10083):1981–2004. doi: 10.1016/S0140-6736(17)30874-7 28433256 10.1016/S0140-6736(17)30874-7 PMC5440770

[61] Wu Y, Duan Z. Analysis on evolution and research focus in psychiatry field. BMC Psychiatry. 2015;15:105 doi: 10.1186/s12888-015-0482-1 25947280 10.1186/s12888-015-0482-1 PMC4464132

[62] Kristiansen CB, Vestergaard CH. Secular trends in the interest of physical health in patients with mental illness. Acta Psychiatr Scand. 2015;132(5):408–10. doi: 10.1111/acps.12474 26366504 10.1111/acps.12474

[63] Teeson M, Proudfoot H. Comorbid mental disorders and substance use disorders: epidemiology, prevention and treatment Sydney: National Drug and Alcohol Research Centre University of New South Wales Sydney; 2004 http://www.health.gov.au/internet/publications/publishing.nsf/Content/drugtreat-pubs-comorbid-toc#cop

[64] Boyd JH, Burke JD Jr, Gruenberg E, Holzer CE 3rd, Rae DS, George LK, et al Exclusion criteria of DSM-III. A study of co-occurrence of hierarchy-free syndromes. Arch Gen Psychiatry. 1984;41(10):983–9. doi: 10.1001/archpsyc.1984.01790210065008 6477056 10.1001/archpsyc.1984.01790210065008

[65] Regier DA, Farmer ME, Rae DS, Locke BZ, Keith SJ, Judd LL, et al Comorbidity of mental disorders with alcohol and other drug abuse. Results from the Epidemiologic Catchment Area (ECA) Study. JAMA. 1990;264:2511–8. doi: 10.1001/jama.1990.03450190043026 2232018

[66] Grant BF, Stinson FS, Dawson DA, Chou SP, Dufour MC, Compton W, et al Prevalence and co-occurrence of substance use disorders and independent mood and anxiety disorders: results from the National Epidemiologic Survey on Alcohol and Related Conditions. Arch Gen Psychiatry. 2004;61:807–16. doi: 10.1001/archpsyc.61.8.807 15289279 10.1001/archpsyc.61.8.807

[67] Lasser K, Boyd JW, Woolhandler S, Himmelstein DU, McCormick D, Bor DH. Smoking and mental illness: A population-based prevalence study. JAMA. 2000;284:2606–10. doi: 10.1001/jama.284.20.2606 11086367 10.1001/jama.284.20.2606

[68] Charlson FJ, Moran AE, Freedman G, Norman RE, Stapelberg NJ, Baxter AJ, et al The contribution of major depression to the global burden of ischemic heart disease: a comparative risk assessment. BMC Med. 2013;11:250 doi: 10.1186/1741-7015-11-250 24274053 10.1186/1741-7015-11-250 PMC4222499

[69] Foguet-Boreu Q, Fernández San Martin MI, Flores Mateo G, Zabaleta Del Olmo E, Ayerbe García-Morzon L, Perez-Piñar López M, et al Cardiovascular risk assessment in patients with a severe mental illness: a systematic review and meta-analysis. BMC Psychiatry. 2016;16:141 doi: 10.1186/s12888-016-0833-6 27176477 10.1186/s12888-016-0833-6 PMC4866037

[70] Batelaan NM, Seldenrijk A, Bot M, van Balkom AJ, Penninx BW. Anxiety and new onset of cardiovascular disease: critical review and meta-analysis. Br J Psychiatry. 2016;208(3):223–31. doi: 10.1192/bjp.bp.114.156554 26932485 10.1192/bjp.bp.114.156554

[71] Catalá-López F, Suárez-Pinilla M, Suárez-Pinilla P, Valderas JM, Gómez-Beneyto M, Martínez S, et al Inverse and direct cancer comorbidity in people with central nervous system disorders: a meta-analysis of cancer incidence in 577,013 participants of 50 observational studies. Psychother Psychosom. 2014;83(2):89–105. doi: 10.1159/000356498 24458030 10.1159/000356498

[72] Catalá-López F, Hutton B, Driver JA, Page MJ, Ridao M, Valderas JM, et al Cancer and central nervous system disorders: protocol for an umbrella review of systematic reviews and updated meta-analyses of observational studies. Syst Rev. 2017;6:69 doi: 10.1186/s13643-017-0466-y 28376926 10.1186/s13643-017-0466-y PMC5379758

[73] Mock CN, Donkor P, Gawande A, Jamison DT, Kruk ME, Debas HT. Essential Surgery: Key Messages of This Volume In: Debas HT, Donkor P, Gawande A, Jamison DT, Kruk ME, Mock CN, editors. Essential Surgery: Disease Control Priorities, Third Edition (Volume 1). Washington (DC): The International Bank for Reconstruction and Development / The World Bank; 2015. 26741007

[74] Gutnik LA, Dielman J, Dare AJ, Ramos MS, Riviello R, Meara JG, et al Funding flows to global surgery: an analysis of contributions from the USA. Lancet. 2015;385 Suppl 2:S51 doi: 10.1016/S0140-6736(15)60846-7 26313101 10.1016/S0140-6736(15)60846-7

[75] Dixon JB, le Roux CW, Rubino F, Zimmet P. Bariatric surgery for type 2 diabetes. Lancet. 2012;379(9833):2300–11. doi: 10.1016/S0140-6736(12)60401-2 22683132 10.1016/S0140-6736(12)60401-2

[76] Keezer MR, Sisodiya SM, Sander JW. Comorbidities of epilepsy: current concepts and future perspectives. Lancet Neurol. 2016;15(1):106–15. doi: 10.1016/S1474-4422(15)00225-2 26549780 10.1016/S1474-4422(15)00225-2

[77] GBD 2015 Neurological Disorders Collaborator Group. Global, regional, and national burden of neurological disorders during 1990–2015: a systematic analysis for the Global Burden of Disease Study 2015. Lancet Neurol. 2017;16(11):877–897. doi: 10.1016/S1474-4422(17)30299-5 28931491 10.1016/S1474-4422(17)30299-5 PMC5641502

[78] Patel V, Chisholm D, Parikh R, Charlson FJ, Degenhardt L, et al Addressing the burden of mental, neurological, and substance use disorders: key messages from Disease Control Priorities, 3rd edition. Lancet. 2016;387:1672–1685. doi: 10.1016/S0140-6736(15)00390-6 26454360 10.1016/S0140-6736(15)00390-6

[79] Jones DS, Podolsky SH, Greene JA. The burden of disease and the changing task of medicine. N Engl J Med. 2012;366(25):2333–8. doi: 10.1056/NEJMp1113569 22716973 10.1056/NEJMp1113569

[80] Kerry VB, Walensky RP, Tsai AC, Bergmark RW, Bergmark BA, Rouse C, et al US medical specialty global health training and the global burden of disease. J Glob Health. 2013;3(2):020406 doi: 10.7189/jogh.03.020406 24363924 10.7189/jogh.03.020406 PMC3868823

[81] Roth GA, Johnson C, Abajobir A, Abd-Allah F, Abera SF, Abyu G, et al Global, Regional, and National Burden of Cardiovascular Diseases for 10 Causes, 1990 to 2015. J Am Coll Cardiol. 2017;70(1):1–25. doi: 10.1016/j.jacc.2017.04.052 28527533 10.1016/j.jacc.2017.04.052 PMC5491406

[82] Global Burden of Disease Cancer Collaboration, Fitzmaurice C, Allen C, Barber RM, Barregard L, Bhutta ZA, et al Global, regional, and national cancer incidence, mortality, years of life lost, years lived with disability, and disability-adjusted life-years for 32 cancer groups, 1990 to 2015: A systematic analysis for the Global Burden of Disease Study. JAMA Oncol. 2017;3(4):524–548. doi: 10.1001/jamaoncol.2016.5688 27918777 10.1001/jamaoncol.2016.5688 PMC6103527

[83] Patel V. Talking sensibly about depression. PLoS Med. 2017;14(4):e1002257 doi: 10.1371/journal.pmed.1002257 28376089 10.1371/journal.pmed.1002257 PMC5380305

[84] Catalá-López F, Moher D, Tabarés-Seisdedos R. Improving transparency of scientific reporting to increase value and reduce waste in mental health research. Rev Psiquiatr Salud Ment. 2016;9(1):1–3. doi: 10.1016/j.rpsm.2016.01.002 26896001 10.1016/j.rpsm.2016.01.002

[85] Patel V, Belkin GS, Chockalingam A, Cooper J, Saxena S, Unützer J. Grand challenges: integrating mental health services into priority health care platforms. PLoS Med. 2013;10(5):e1001448 doi: 10.1371/journal.pmed.1001448 23737736 10.1371/journal.pmed.1001448 PMC3666874

[86] Bolton P, Lee C, Haroz EE, Murray L, Dorsey S, et al A transdiagnostic community-based mental health treatment for comorbid disorders: development and outcomes of a randomized controlled trial among Burmese refugees in Thailand. PLoS Med. 2014;11: e1001757 doi: 10.1371/journal.pmed.1001757 25386945 10.1371/journal.pmed.1001757 PMC4227644

[87] Muth C, Glasziou PP. Guideline recommended treatments in complex patients with multimorbidity. BMJ. 2015;351:h5145 doi: 10.1136/bmj.h5145 26431846 10.1136/bmj.h5145

[88] Guthrie B, Payne K, Alderson P, McMurdo ME, Mercer SW. Adapting clinical guidelines to take account of multimorbidity. BMJ. 2012;345:e6341 doi: 10.1136/bmj.e6341 23036829 10.1136/bmj.e6341

[89] Jadad AR, To MJ, Emara M, Jones J. Consideration of multiple chronic diseases in randomized controlled trials. JAMA. 2011;306(24):2670–2. doi: 10.1001/jama.2011.1886 22203536 10.1001/jama.2011.1886

[90] Fortin M, Smith SM. Improving the external validity of clinical trials: the case of multiple chronic conditions. J Comorb. 2013;3:30–35. doi: 10.15256/joc.2013.3.27 29090144 10.15256/joc.2013.3.27 PMC5636029

[91] Mercer SW, Guthrie B, Furler J, Watt GC, Hart JT. Multimorbidity and the inverse care law in primary care. BMJ. 2012;344:e4152 doi: 10.1136/bmj.e4152 22718915 10.1136/bmj.e4152

[92] Page MJ, Shamseer L, Altman DG, Tetzlaff J, Sampson M, Tricco AC, et al Epidemiology and reporting characteristics of systematic reviews of biomedical research: A cross-sectional study. PLoS Med. 2016;13(5):e1002028 doi: 10.1371/journal.pmed.1002028 27218655 10.1371/journal.pmed.1002028 PMC4878797

[93] Neufeld NH, Sharma B, McGirr A. Debates in medicine: global representation in medical discourse. Lancet. 2014;383(9919):779 doi: 10.1016/S0140-6736(14)60396-2 24581659 10.1016/S0140-6736(14)60396-2

