# Supplementary material for: Mapping of global scientific research in comorbidity and multimorbidity: A cross-sectional analysis
Source: PLoS One. 2018 Jan 3;13(1):e0189091. doi: 10.1371/journal.pone.0189091 (PMC5751979; doi:10.1371/journal.pone.0189091)
Supplement: S1 Table — (DOCX) [file pone.0189091.s002.docx]

**S1 Table. Search strategy and results.**

|  | **Terms (“topic”)** | **Results** |
| --- | --- | --- |
| # 1 | multidisease* | 31 |
| # 2 | multi-disease* | 64 |
| # 3 | comorbidit* | 74145 |
| # 4 | co-morbidit* | 12828 |
| # 5 | multimorbidit* | 1633 |
| # 6 | multi-morbidit* | 227 |
| # 7 | multipatholog* | 4 |
| # 8 | multi-patholog* | 6 |
| # 9 | pluripatholog* | 11 |
| # 10 | pluri-patholog* | 0 |
| # 11 | polipatholog* | 0 |
| # 12 | poli-pathology* | 0 |
| # 13 | polimorbidit* | 0 |
| # 14 | poli-morbidit* | 0 |
| # 15 | #14 OR #13 OR #12 OR #11 OR #10 OR #9 OR #8 OR #7 OR #6 OR #5 OR #4 OR #3 OR #2 OR #1 | 85994 |
| Limits | Type of document: (Article OR Review) |  |
